# An experimental study of strong reciprocity in bacteria

**DOI:** 10.1098/rsbl.2013.1069

**Published:** 2014-02

**Authors:** R. Fredrik Inglis, Stuart West, Angus Buckling

**Affiliations:** 1 Department of Environmental Sciences, Eidgenössiche Technische Hochschule Zürich, Zürich 8092, Switzerland; 2 Department of Environmental Microbiology, Swiss Federal Institute of Aquatic Science and Technology (Eawag), PO Box 611, Dübendorf 8600, Switzerland; 3 Department of Zoology, University of Oxford, Oxford OX1 3PS, UK; 4 Department of Biosciences, University of Exeter, Penryn TR10 9EZ, UK

**Keywords:** *Pseudomonas aeruginosa*, bacteriocin, siderophore

## Abstract

Strong reciprocity, whereby cooperators punish non-cooperators, may help to explain the evolutionary success of cooperative behaviours. However, theory suggests that selection for strong reciprocity can depend upon tight genetic linkage between cooperation and punishment, to avoid the strategy being outcompeted by non-punishing cooperators. We tested this hypothesis using experimental populations of the bacterium *Pseudomonas aeruginosa*, which cooperate by producing iron-scavenging siderophores and, in this context, punish non-cooperators with toxins. Consistent with theory, we show that cooperative punishers can indeed invade cheats, but only when the traits are tightly linked. These results emphasize that punishment is only likely to be favoured when the punishment itself leads to a direct or indirect fitness benefit to the actor.

## 1. Introduction

The evolution of cooperation in humans has been argued to have been facilitated by strong reciprocity: helping other cooperators while punishing individuals who do not cooperate [1–6]. However, just as cooperation can be invaded by non-cooperators (in the absence of benefits of cooperation), cooperative non-punishers can invade cooperative punishers. In other words, invoking punishment to explain altruistic cooperation is simply deferring the problem of cooperation to another trait [7]. Lehmann *et al*. [8] argued that a key factor in some analyses was the assumption that cooperation and punishment were genetically linked, behaving as a single Mendelian trait [8]. In this case, harming is also directed at non-harmers, and strong reciprocity can then readily invade from rare in a structured population of defectors [8]. By contrast, if the traits are unlinked, then neither strong reciprocity nor cooperation by itself can invade the population, unless other factors are incorporated, such as individuals adjusting conditionally whether they cooperate or defect [9].

In this study, we use experimental populations of the bacterium *Pseudomonas aeruginosa* to test the predicted role of tight linkage between cooperation and punishment in the invasion of strong reciprocity. Specifically, we examine a scenario where cooperation and cheating are fixed rather than conditional behaviours, in which case we predict that cooperation and punishment can only invade when they are tightly linked. *Pseudomonas aeruginosa*, and bacteria in general, have been shown to engage in numerous social interactions such as the cooperative production of siderophores (iron-scavenging molecules that can be considered public goods) [10,11] and the ability to kill closely related strains through production of anti-competitor toxins, for example bacteriocins [12,13]. A co-expression of cooperative and harming traits would be analogous to strong reciprocity, if bacteria were able to preferentially harm individuals that do not engage in the cooperative act. This can be the case in *P. aeruginosa*, as many bacteriocins and siderophores are transported into the cell via the same cell-surface receptor (fpvA), which leads to a ‘functional’ linkage between the two traits [14]. Bacterial cells that are able to take up siderophores but do not produce them (i.e. ‘cheats’) could then be killed by bacteriocins made by the siderophore-producing strain, while the bacteriocin- and siderophore-producing strain remains unharmed owing to the co-expression of an immunity compound encoded in the bacteriocin operon [15].

We created replicate metapopulations of bacteriocin-sensitive, non-siderophore-producing cheats and tested whether these could be invaded by: (i) a wild-type strain of *P. aeruginosa* (PAO1) that can be considered a ‘strong reciprocator’ as it produces both cooperative siderophores (pyoverdine type I) and harming bacteriocins (pyocin S2); (ii) an isogenic bacteriocin-knock-out mutant (PAO1150-2) that can be considered a non-punishing cooperator as it only produces cooperative siderophores and (iii) a 1 : 1 mixture of both strains (PAO1 and PAO1150-2), in order to mimic a break in genetic linkage between the cooperating and punishing trait. We predict that only in the first scenario will siderophore producers successfully invade.

## 2. Material and methods

### (a) Bacterial strains

We used *P. aeruginosa* strain PAO1 as a strong reciprocator (cooperative punisher), as it is a known producer of pyocin S2 (punishing behaviour) and pyoverdine type I (cooperative behaviour). We used PAO1150-2, a transposon knock-out mutant of the *psy2* gene involved in S2 production (not spiteful), which still produces pyoverdine type I (cooperative behaviour), as a cooperative non-punisher. These strains were competed against six clones of serotype O:9 (cheats) which are sensitive to S2 pyocins and show a reduction in iron-chelating ability by over 60% [16]. Strains were grown in 30 ml glass universals containing 6 ml of King's medium B (KB), shaking at 0.65 g and 37°C and were subsequently diluted to equal densities to start the experiment.

### (b) Experimental design

In this experiment, we set out to test the ability of cooperators to invade a population of cheats. To facilitate this, we performed three different treatments: (i) a cooperative strain (PAO1150-2) invading a population of cheats (O:9), (ii) a cooperative strain that also produces a punishing toxin (PAO1) invading a population of cheats (O:9) and (iii) a cooperative strain (PAO1150-2) and a cooperative strain that produces a punishing toxin (PAO1) invading a population of cheats (O:9). We replicated each treatment in six metapopulations that contain nine individual subpopulations. Each subpopulation was grown in a tube of KB broth.

At the start of the experiment, every subpopulation was inoculated with approximately 10 000 cells of O:9. One subpopulation within each metapopulation was inoculated by 1% of the invading strain (circa 100 cells) of either PAO1150-2 (treatment 1), PAO1 (treatment 2) and a 1 : 1 mixture of PAO1150-2 and PAO1 (a 0.5% starting inoculum of each strain) (treatment 3). Although in our last treatment (treatment 3), we only added 0.5% of PAO1 and PAO1150-2, previous studies have shown that when starting at lower frequencies (e.g. 0.1%) both strains display a marked increase in fitness when competed against O:9 cheats and can be directly compared to 1% starting inocula [16]. Cultures were then grown at 37°C for 96 h in an orbital incubator, shaking at 0.65 g. Every tube, within a metapopulation, was then subsequently mixed together by adding equal volumes of culture. This mixture (one for each metapopulation, six for each treatment) was diluted and then transferred to nine fresh tubes of KB, with approximately 10 000 cells being transferred each time. This selection procedure was repeated for a further eight transfers (approx. 70 bacterial generations). At every round of selection, we scored the frequencies of the strains (based on their colony morphology and different antibiotic resistance profiles) by growing them on agar plates and counting colony forming units, measuring the time at which the invading strains either went extinct or to fixation. We performed a parametric survival analysis with censoring (as not all invading strains went extinct) and a Weibull error distribution on the data using R v. 3.0.2.

## 3. Results and discussion

**Figure 1.**
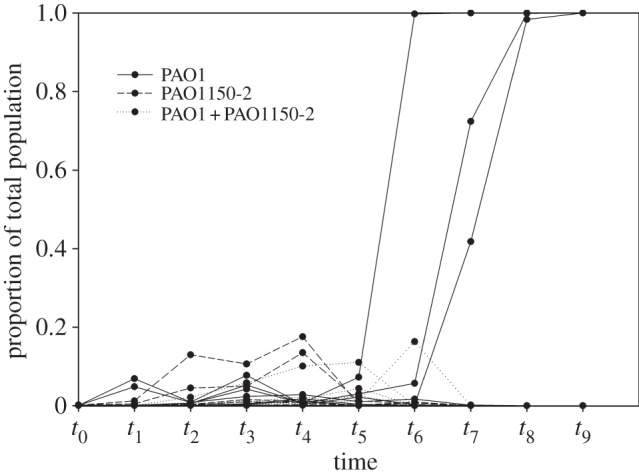
Invasion of cooperating punishers in a population of public goods cheats when initially rare. Only when cooperation and punishment are in linkage is the strain (PAO1) able to invade. A solely cooperating genotype (PAO1150-2) is unable to invade, and when the linkage is broken (through a process such as recombination) the strain is no longer able to invade (PAO1 and PAO1150-2).

We empirically investigated the importance of linkage between cooperation (siderophore production) and punishment (bacteriocin production) in determining the invasion of strong reciprocity (punishing cooperators) in the bacterium *P. aeruginosa*. Consistent with theory [8], we found that only when cooperation and punishment traits were in tight linkage were cooperators able to invade (in three out of six populations); in all other populations, cooperators were driven to extinction (one-tailed Fisher's exact test, *p* = 0.037). To take into account quantitative differences between replicates, we compared extinction times between treatments. This did not differ between our non-punishing cooperator and the mix of both strains (*z* = 1.14, *p* > 0.25), whereas extinction occurred more rapidly in both non-punishing cooperators (*z* = 5.56, *p* < 0.001) and the mix of both strains (*z* = 4.45, *p* < 0.001) than the punishing cooperator (figure 1).

The cooperative punishing strain was able to increase in frequency by selectively killing sensitive cheats, thereby reducing the local intensity of competition. However, this invasion was clearly not deterministic as it only occurred in three out of six metapopulations (figure 1). Invasion of individually costly toxin production (or harming behaviours in general) is positive-frequency dependent, because at very low frequencies harming toxin will benefit susceptibles as much as producers [12]. Presumably, through stochastic events, the number of toxin producers in some patches in the metapopulation exceeded the threshold where the toxin will benefit producers more than the susceptibles. Patch population size would have increased with increasing frequency of siderophore producers [11], further facilitating the spread of punishing cooperators [8]. Furthermore, because the number of subpopulations is limited in our metapopulation structure, drift can have an appreciable influence on the evolution of cooperation or punishment [17].

Breaking up the linkage between cooperation and punishment prevented the invasion of the cooperators because non-toxin producers, which do not pay the cost of toxin production and are themselves resistant to toxin, would have outcompeted the toxin producers. In turn, the non-punishing cooperators would have then been outcompeted by the siderophore cheats, as shown by the failure of the non-punishing cooperator by itself to invade populations of siderophore cheats. This proposed scenario bears some resemblance to the rock–paper–scissors interactions previously observed for bacteriocin-producing strategies, (where producers kill sensitives but resistant non-producers outcompete producers, which are then in turn outcompeted by sensitives [18–20]), but in this case cooperative interactions are also present.

Our results show that cooperation and punishment can be favoured when they are genetically linked. However, there is no reason to expect that this will generally be the case. For example, in humans, both these traits will be controlled by multiple genes and there is no reason to expect that both traits will be controlled by the same genes or closely linked genes [21]. What matters here is not whether the genes can become linked, but rather do they have to be linked—if they do not have to be linked, then individuals who do not punish could readily invade. Nonetheless, there are scenarios where punishment can be favoured when it is not genetically linked to cooperation [8,9,22].

Here, we artificially created a situation of strong reciprocity (i.e. by employing susceptible cheats), but natural associations between pyocins and pyoverdine suggest that strong reciprocity may occur in natural populations of *P. aeruginosa* and perhaps bacteria in general. Specifically, the punishing trait (pyocin S2) and cooperative trait (type 1 pyoverdine) share a common receptor (fpvA) leading to a ‘functional’ linkage between these traits [14], as is the case for other pyoverdine–bacteriocin combinations [23]. Interestingly, bacteriocin diversity may help to explain the observed diversity in pyoverdine types and associated receptors [24], because susceptibility to a bacteriocin would impose strong selection to alter to both the receptor and pyoverdine. This type of balancing selection has previously been implicated in maintaining diversity in the context of host–parasite interactions [25].

## Data accessibility

All data have been deposited in dryad: doi:10.5061/dryad.cs31j.

## Funding statement

We thank the Natural Environment Research Council, The Royal Society and the European Research Council for financial support.

## References

[1] Gintis H 2000 Strong reciprocity and human sociality. J. Theor. Biol. 206, 169–179 (doi:10.1006/jtbi.2000.2111) 10966755 10.1006/jtbi.2000.2111

[2] Gintis H 2003 Solving the puzzle of prosociality. Ration. Soc. 15, 155–187 (doi:10.1177/1043463103015002001)

[3] Fehr E Fischbacher U 2003 The nature of human altruism. Nature 425, 785–791 (doi:10.1038/nature02043) 14574401 10.1038/nature02043

[4] Gintis H Bowles S Boyd R Fehr E 2003 Explaining altruistic behavior in humans. Evol. Hum. Behav. 24, 153–172 (doi:10.1016/S1090-5138(02)00157-5)

[5] Boyd R Gintis H Bowles S Richerson PJ 2003 The evolution of altruistic punishment. Proc. Natl Acad. Sci. USA 100, 3531–3535 (doi:10.1073/pnas.0630443100) 12631700 10.1073/pnas.0630443100 PMC152327

[6] Bowles S Gintis H 2004 The evolution of strong reciprocity: cooperation in heterogeneous populations. Theor. Popul. Biol. 65, 17–28 (doi:10.1016/j.tpb.2003.07.001) 14642341 10.1016/j.tpb.2003.07.001

[7] Gardner A West SA 2004 Cooperation and punishment, especially in humans. Am. Nat. 164, 753–764 (doi:10.1086/425623) 10.1086/425623 29641920

[8] Lehmann L Rousset F Roze D Keller L 2007 Strong reciprocity or strong ferocity? A population genetic view of the evolution of altruistic punishment. Am. Nat. 170, 21–36 (doi:10.1086/518568) 17853989 10.1086/518568

[9] Boyd R Gintis H Bowles S 2010 Coordinated punishment of defectors sustains cooperation and can proliferate when rare. Science 328, 617–620 (doi:10.1126/science.1183665) 20431013 10.1126/science.1183665

[10] West SA Buckling A 2003 Cooperation, virulence and siderophore production in bacterial parasites. Proc. R. Soc. Lond. B 270, 37–44 (doi:10.1098/rspb.2002.2209) 10.1098/rspb.2002.2209 PMC1691207 12590769

[11] Griffin AS West SA Buckling A 2004 Cooperation and competition in pathogenic bacteria. Nature 430, 1024–1027 (doi:10.1038/nature02744) 15329720 10.1038/nature02744

[12] Gardner A West SA Buckling A 2004 Bacteriocins, spite and virulence. Proc. R. Soc. Lond. B 271, 1529–1535 (doi:10.1098/rspb.2004.2756) 10.1098/rspb.2004.2756 PMC1691756 15306326

[13] Inglis RF Gardner A Cornelis P Buckling A 2009 Spite and virulence in the bacterium *Pseudomonas aeruginosa*. Proc. Natl Acad. Sci. USA 106, 5703–5707 (doi:10.1073/pnas.0810850106) 19321425 10.1073/pnas.0810850106 PMC2667014

[14] Denayer S Matthijs S Cornelis P 2007 Pyocin S2 (Sa) kills *Pseudomonas aeruginosa* strains via the FpvA type I ferripyoverdine receptor. J. Bacteriol. 189, 7663–7668 (doi:10.1128/JB.00992-07) 17720787 10.1128/JB.00992-07 PMC2168733

[15] Michel-Briand Y Baysse C 2002 The pyocins of *Pseudomonas aeruginosa*. Biochimie 84, 499–510 (doi:10.1016/S0300-9084(02)01422-0) 12423794 10.1016/s0300-9084(02)01422-0

[16] Inglis RF Brown SP Buckling A 2012 Spite versus cheats: competition among social strategies shapes virulence in *Pseudomonas aeruginosa*. Evolution 66, 3472–3484 (doi:10.1111/j.1558-5646.2012.01706.x) 23106711 10.1111/j.1558-5646.2012.01706.x PMC3795443

[17] Kummerli R Gardner A West SA Griffin AS 2009 Limited dispersal, budding dispersal, and cooperation: an experimental study. Evolution 63, 939–949 (doi:10.1111/j.1558-5646.2008.00548.x) 19154373 10.1111/j.1558-5646.2008.00548.x

[18] Kerr B Riley MA Feldman MW Bohannan BJM 2002 Local dispersal promotes biodiversity in a real-life game of rock–paper–scissors. Nature 418, 171–174 (doi:10.1038/nature00823) 12110887 10.1038/nature00823

[19] Czárán TL Hoekstra RF 2003 Killer-sensitive coexistence in metapopulations of micro-organisms. Proc. R. Soc. Lond. B 270, 1373–1378 (doi:10.1098/rspb.2003.2338) 10.1098/rspb.2003.2338 PMC1691387 12965028

[20] Biernaskie JM Gardner A West SA 2013 Multicoloured greenbeards, bacteriocin diversity and the rock–paper–scissors game. J. Evol. Biol. 26, 2081–2094 (doi:10.1111/jeb.12222) 23980628 10.1111/jeb.12222

[21] West SA Gardner A 2010 Altruism, spite, and greenbeards. Science 327, 1341–1344 (doi:10.1126/science.1178332) 20223978 10.1126/science.1178332

[22] Clutton-Brock TH Parker GA 1995 Punishment in animal societies. Nature 373, 209–216 (doi:10.1038/373209a0) 7816134 10.1038/373209a0

[23] de Chial M 2003 Identification of type II and type III pyoverdine receptors from *Pseudomonas aeruginosa*. Microbiology 149, 821–831 (doi:10.1099/mic.0.26136-0) 12686625 10.1099/mic.0.26136-0

[24] Meyer JM Stintzi A De Vos D Cornelis P Tappe R Taraz K Budzikiewicz H 1997 Use of siderophores to type pseudomonads: the three *Pseudomonas aeruginosa* pyoverdine systems. Microbiology 143, 35–43 (doi:10.1099/00221287-143-1-35) 9025276 10.1099/00221287-143-1-35

[25] Haldane JBS 1949 Disease and evolution. Ric. Sci. Suppl. A 19, 68–76

